# An improved equilibrium optimization algorithm for feature selection problem in network intrusion detection

**DOI:** 10.1038/s41598-024-67488-7

**Published:** 2024-08-12

**Authors:** Zahra Asghari Varzaneh, Soodeh Hosseini

**Affiliations:** Department of Computer Science, Faculty of Mathematics and Computer, Shahid Bahonar University of Kerman, Kerman, Iran

**Keywords:** Equilibrium optimizer, Feature selection, Levy flight, Opposition-based learning, Intrusion detection system, Engineering, Mathematics and computing

## Abstract

In this paper, an enhanced equilibrium optimization (EO) version named Levy-opposition-equilibrium optimization (LOEO) is proposed to select effective features in network intrusion detection systems (IDSs). The opposition-based learning (OBL) approach is applied by this algorithm to improve the diversity of the population. Also, the Levy flight method is utilized to escape local optima. Then, the binary rendition of the algorithm called BLOEO is employed to feature selection in IDSs. One of the main challenges in IDSs is the high-dimensional feature space, with many irrelevant or redundant features. The BLOEO algorithm is designed to intelligently select the most informative subset of features. The empirical findings on NSL-KDD, UNSW-NB15, and CIC-IDS2017 datasets demonstrate the effectiveness of the BLOEO algorithm. This algorithm has an acceptable ability to effectively reduce the number of data features, maintaining a high intrusion detection accuracy of over 95%. Specifically, on the UNSW-NB15 dataset, BLOEO selected only 10.8 features on average, achieving an accuracy of 97.6% and a precision of 100%.

## Introduction

Computer networks have become a fundamental aspect of our daily lives, from business operations to personal communication. However, the growing utilization of computer networks has also resulted in a rise in network attacks, making network security a top priority^1,2^. Network attacks can result in data theft, system damage, and financial losses. Therefore, it is essential to have a robust IDS in place that can identify and prevent unauthorized access to the system^3^. Therefore, network security is of paramount importance in today's Internet-connected world. Intrusion detection systems are key tools for protecting networks and detecting potential threats^4^. One of the techniques that can be effectively used to detect network intrusion using a trained dataset of network attacks is machine learning. The data set extracted from the network can include various features such as network traffic, network resource usage, and user activities, which are used to describe an instance in the data set^5,6^.

However, IDSs face the challenge of high-dimensional data containing many redundant or irrelevant features^7^. Feature selection that identifies a minimal set of important features, can enhance the performance of IDSs by reducing dimensionality, enhancing accuracy, and speeding up the learning process. Feature selection is performed to enhance the model's execution by decreasing the complexity and the dimensionality of the input space and to avoid overfitting^8,9^. Relevancy measures the degree of a feature's relationship with the target variable. Relevance features are more likely to be useful for predicting the target variable. On the other hand, when there are two or more features with the same information in the data set, data redundancy occurs and causes the machine learning model to learn noise in the data instead of basic patterns, which leads to overfitting and cannot be good to generalize on new data. In the feature selection process, a subset of features is identified from the dataset to reduce the amount of noise and data redundancy for use in machine learning^10,11^.

There are several techniques for selecting features, such as filter, wrapper, and embedded approaches^12^. The top-ranked features are chosen using filter methods, which rank the features according to how relevant they are to the target variable. Using several feature subsets, wrapper techniques assess the model's performance and choose the subset with the best performance. Feature selection is a step in the training of a model in embedded techniques, such as regularization techniques in linear models^13^.

Wrapper approaches examine the space of potential feature subsets using a search algorithm, like a metaheuristic algorithm and select the subset that gives the best performance^14^. In wrapper methods for feature selection, a metaheuristic algorithm is utilized to search for the ideal selection of features to optimize the model's performance. The algorithm assesses the model's performance using a validation set and identifies the subset of features that results in superior performance. This process is repeated multiple times with various subsets of features, and the subset that delivers the optimal performance overall is chosen as the final set of features^15^. Optimization techniques known as metaheuristics are created to identify the best answers to challenging optimization issues^16,17^. These algorithms use a random search mechanism to explore the solution space and make use of heuristics to direct the search toward areas of space that show promise^18,19^. They are often used for feature selection because they can efficiently search through large search spaces and find near-optimal solutions^20,21^.

These algorithms can explore different combinations of features and evaluate their performance without exhaustively searching through all possible feature subsets, which can require substantial computational resources or even be infeasible for large datasets^22^. Several metaheuristic algorithms have been successfully applied to feature selection, which requires the identification of a subset of pertinent features from a high-dimensional dataset. Some metaheuristic algorithms for feature selection include Particle Swarm Optimization (PSO)^23^, Grey Wolf Optimizer (GWO)^24^, Horse herd Optimization Algorithm (HOA)^25^, Starling Murmuration Optimizer(SMO)^26^, Harris hawks optimizer (HHO)^27^, Harmony search algorithm (HS)^28^, and Differential Evolution (DE)^29^. These algorithms have been shown to effectively pick the most informative features while decreasing the problem's computational complexity. In the last few years, a metaheuristic algorithm called the equilibrium optimization (EO) algorithm was introduced and inspired by physics^30^. The most important reason for choosing the EO algorithm was to provide very competitive results compared to existing powerful algorithms in solving complex problems with high dimensions such as network intrusion detections. In this paper, the primary issue is feature selection to reduce data dimensions. As a result, the EO algorithm was selected due to its strong global search capabilities, resilience to local optima, and computational efficiency. These factors are all crucial considerations for effective feature selection. Also, simplicity in the execution and implementation of the problem in the EO was another reason for choosing this algorithm. However, like other metaheuristic algorithms, the EO algorithm exhibits some limitations including slow convergence and falling into local optima^31^. In this study, we proposed an enhanced EO algorithm named BLOEO for selecting effective features in intrusion detection. The BLOEO algorithm incorporates Opposition-based Learning to improve the diversity of the population and the Levy flight mechanism to escape local optima. In the following, the study's contributions are briefly stated.

- Presenting a novel feature selection method, employing an improved binary EO.
- Improving the diversity of individuals in the population and improving the exploration phase of the EO algorithm by using Opposition-based Learning and employing the levy flight to escape from the local optimum.
- Detection of network intrusions by selecting optimal features, and proposing BLOEO algorithm.
- Evaluating the efficiency of BLOEO using NSL-KDD, UNSW-NB15, and CIC-IDS2017 datasets and comparing the test findings with other metaheuristic algorithms concerning accuracy, recall, specificity, precision, and F-Score.

This paper is organized as follows: Section "Related work" gives a brief review of the related works. Section "Equilibrium optimizer" outlines the standard EO algorithm. Section "Proposed algorithm" includes the details of the proposed algorithm, LOEO. The simulation and results of intrusion detection datasets in the feature selection issue are provided in Sect. "Experimental results". Finally, Sect. "Conclusion and future works" contains the conclusions and future direction of the study.

## Related work

The issue of network security has become increasingly important as computer networks are being used in various fields. An intrusion detection system's objective is to determine and avoid unauthorized entry into the system. However, the existence of a vast number of features in IDSs poses a challenge. To address this challenge, researchers have proposed multiple feature selection algorithms for IDSs. These algorithms aim to identify the most useful and effective features from the data to enhance the accuracy and efficiency of the IDS.

ZHAO et al.^32^ introduced a new IDS that combines feature selection with a weighted stacking classifier named CFS-DE, to constrain the dimension of the features, and enhance the classification performance. CFS-DE is used to search for the most suitable set of features, Meanwhile, the weighted stacking algorithm improves the base classifier weights that exhibit favorable training results and reduces the weights of those with unfavorable results. The system aims to enhance the efficiency of intrusion detection by decreasing the dimension of features and enhancing the accuracy of the classification. Hajisalem and Babaei^33^ proposed a novel hybrid classification approach that integrates two optimization algorithms ABC and AFS. The approach incorporates Correlation-based Feature Selection and Fuzzy C-means clustering methods to partition the training dataset and eliminate irrelevant features. To differentiate between normal and anomalous records, their method uses the CART to build If–Then rules based on the selected attributes. Asghari Varzaneh et al.^34^ introduced a fuzzy rule-based classification framework to detect intrusions within computer network environments. To bolster the classification efficacy, the researchers devised a novel technique relying on Genetic Algorithms (GA) to optimize the rule weighting scheme. The proposed methodology was validated using the benchmark KDD99 dataset, and the experimental findings indicate that it significantly improves the detection accuracy and reduces the false alarm rate of the fuzzy rule-based classification system. Samadi Bonab et al.^35^ introduced a method to detect the most important features for constructing an IDS and proposed a new hybrid method based on FFA and ALO optimization algorithms to identify the optimal features and improve the performance of IDS. The proposed method is intended to enhance the effectiveness of IDS by identifying important features from a high-dimensional dataset. Emary et al.^36^ proposed a binary variant of the ALO algorithm specifically designed for wrapper-based feature selection. They utilized a K-Nearest Neighbors (KNN) classifier and aimed to discover an ideal subset of features that maximizes classification performance. The proposed method was performed on 21 standard datasets concerning evaluation criteria. In^37^, a wrapper-based model was proposed using an adapted whale optimization algorithm (WOA) for intrusion detection. To overcome the issue of early convergence resulting in a local optimal solution, the authors hybrid WOA with operators of the genetic algorithm. The suggested method uses the SVM algorithm to find important features in network data to accurately identify intrusions.

Alazzam et al.^38^ developed a feature selection method for IDS that employs the PIO for the selection process. The authors also proposed a novel model for binarizing a continuous PIO and compared it to the traditional ways. The developed model aims to enhance the performance of IDS by selecting the most important features from a high-dimensional dataset. Al-Yaseen et al.^39^ proposed an optimized wrapper feature selection method to boost the efficiency and decrease the processing time of IDS. The method selects relevant features based on a differential evaluation algorithm and then assesses the features utilizing a classifier. Fatani et al.^40^ developed new techniques for IDS feature extraction and selection using swarm intelligence algorithms. The authors designed a mechanism for extracting features with convolutional neural networks (CNN) and presented an alternative feature selection approach using the Aquila optimizer (AQU). The introduced approach aims to improve the effectiveness of IDS by identifying the best features from a high-dimensional dataset.

In^41^, the researchers developed an intrusion detection model that makes use of an enhanced Random Forest (RF) classifier and BMRF optimization employing an adaptive S-shape operation. The RF classifier is applied for feature evaluation and to construct a model for intrusion detection, while the BMRF method is applied to determine which features from intrusion detection datasets are most relevant and eliminate redundant and unnecessary ones. Otair et al.^42^ proposed an enhanced GWO-based PSO for IDSs in wireless sensor networks. The proposed technique utilizes the GWO algorithm for feature selection and hybridizes it with PSO to incorporate the most advantageous data for every gray wolf position using the best value. The PSO algorithm preserves the individual's best position information to avoid the GWO from getting trapped in a local optimum.

One of the techniques that can be effectively used to detect network intrusion using a trained dataset of network attacks is machine learning. The data set extracted from the network can include various features such as network traffic, network resource usage, and user activities, which are used to describe an instance in the data set.

## Equilibrium optimizer

Faramarzi et al.^30^ introduced a novel metaheuristic algorithm in 2020, based on physics, and for each optimization issue, it predicts equilibrium states as the best solution using a model of dynamic mass balance on a control volume. The EO consists of an initial population of concentration vectors in the search space, where every vector depicts a possible fix and is treated as its position. The initial population is generated using the following formula to begin the optimization process:

1 $${C}_{i}^{d}= LB+{rand}_{i}^{d}\times \left(UB-LB\right), i=\text{1,2}, \dots , N and d=\text{1,2}, \dots , D$$

where the population's size is established by N, the size of the problem's dimensions is indicated by D, the lower bound by *LB* and the upper bound by *UB*, and the initial concentration vector of the *i*th individual candidate in the population is represented by ${C}_{i}^{d}$. The vector ${rand}_{i}^{d}$ is in the range of [0,1].

The EO algorithm converges to an equilibrium state, which represents the outcome of the optimization process. However, only equilibrium candidates are utilized to direct the individual in their search pattern; the final equilibrium balance remains unknown. The four top individuals identified in EO by their fitness scores make up the equilibrium candidates, which are meant to increase the capacity for exploration. To encourage better exploitation, the average of the top four individuals is also presented. The vector that contains these five equilibrium candidates is called the equilibrium pool, and it has the following definition.

2 $${\overrightarrow{C}}_{eq,pool}=\left\{{\overrightarrow{C}}_{eq(1)}, {\overrightarrow{C}}_{eq(2)},{\overrightarrow{C}}_{eq(3)}, {\overrightarrow{C}}_{eq(4)},{\overrightarrow{C}}_{eq(avg)}\right\} ,$$

where,

3 $${\overrightarrow{C}}_{eq(avg)}=\frac{{\overrightarrow{C}}_{eq\left(1\right)}+ {\overrightarrow{C}}_{eq\left(2\right)}+{\overrightarrow{C}}_{eq\left(3\right)}+ {\overrightarrow{C}}_{eq\left(4\right)}}{4}$$

and

4 $${f}_{{C}_{eq(1)}}\le {f}_{{C}_{eq\left(2\right)}}\le {f}_{{C}_{eq\left(3\right)}}\le {f}_{{C}_{eq\left(4\right)}} ,$$

where vector ${\overrightarrow{C}}_{eq,pool}$ determines the equilibrium pool, ${\overrightarrow{C}}_{eq(1)}, {\overrightarrow{C}}_{eq(2)},{\overrightarrow{C}}_{eq(3)}, {\overrightarrow{C}}_{eq(4)}$ are the top four candidates identified thus far, and the average of the top four candidates is ${\overrightarrow{C}}_{eq(avg)}$. In each iteration, utilizing the same probability for random selection among potential solutions, the concentration of individuals is updated. Equation (5) is used to update the concentration vectors:

5 $${C}_{new}={C}_{eq}+\frac{G}{\lambda }\left(1-F\right)+\left({C}_{old}-{C}_{eq}\right)\times F ,$$

where ${C}_{old}$ and ${C}_{new}$ denote the present concentration and the new concentration vectors of individuals, respectively. In the equilibrium pool, one concentration vector is arbitrarily chosen ${C}_{eq}$. Equation (6) is used to calculate the vector F, often determined as the exponential term:

6 $$\overrightarrow{F}={e}^{-\overrightarrow{\lambda }(t-{t}_{0})} ,$$

where λ is a random vector with d dimensions, in the range of 0–1. With each iteration increment is calculated as follows—where $Iter$ is the present iteration and $Max\_\text{Iter}$ is the maximum iteration—the *t* parameter is lowered.

7 $$t={(1-\frac{\text{Iter}}{Max\_iter})}^{({a}_{2}\frac{\text{Iter}}{Max\_iter})} ,$$

where the capacity to exploit is controlled by ${a}_{2}$. The ${a}_{2}$ variable in the EO algorithm is set to 1. The value of the *t*_*0*_ is calculated with Eq. (8) and it controls the exploration and exploitation, where *r* is identified with a random vector in the range of 0 and 1, $sign\left(\overrightarrow{r}-0.5\right)$ show the orientation of exploitation and exploration during the search process.

8 $${\overrightarrow{t}}_{0}=\frac{1}{\overrightarrow{\lambda }}\text{ln}\left(-{a}_{1}sign\left(\overrightarrow{r}-0.5\right)\left[1-{e}^{-\overrightarrow{\lambda }t}\right]\right)+t$$

*a*_*1*_ is constant number and controls the exploration capability and its value is 1. The final variant of the exponential is obtained by substituting Eq. (8) into Eq. (6):

9 $$\overrightarrow{F}={a}_{1}sign\left(\overrightarrow{r}-0.5\right)\left[{e}^{-\overrightarrow{\lambda }t}-1\right] .$$

**Figure 1:**
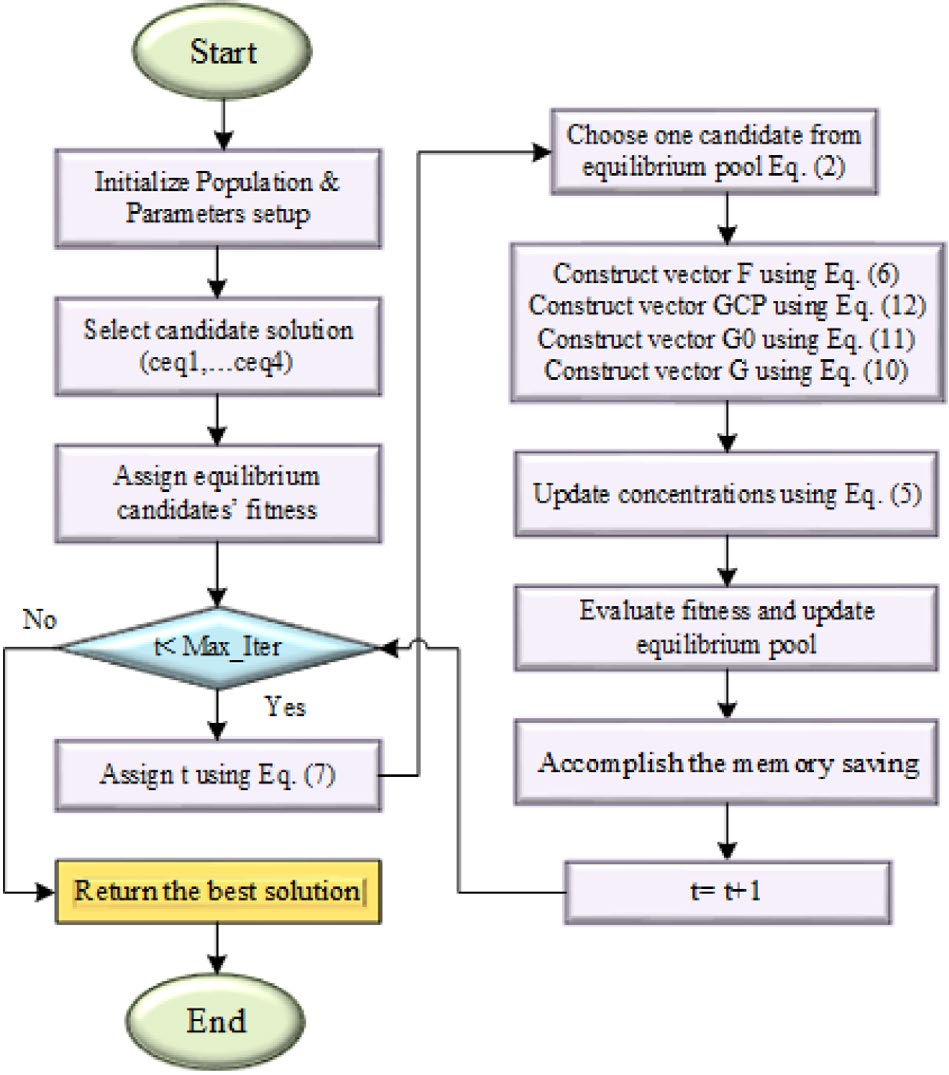
Flowchart of the equilibrium optimizer.

One of the key factors in the EO influencing the exploitation capabilities is the generation rate *G*. The calculation for this parameter is as follows:

10 $$\overrightarrow{G}= {\overrightarrow{G}}_{0}{e}^{-\overrightarrow{\lambda }(t-{t}_{0})}= {G}_{0}F ,$$

where,

11 $$\overrightarrow{{G}_{0}}=\overrightarrow{GCP}\left(\overrightarrow{{C}_{eq}}-\overrightarrow{\lambda }\overrightarrow{C}\right)$$

12 $$\overrightarrow{GCP}=\left\{\begin{array}{c}0.5 {r}_{1 } {r}_{2}\ge GP \\ 0 { r}_{2}<GP \end{array},\right.$$

where the random values in [0,1] are *r*_*1*_ and *r*_*2*_. The *GCP* vector is to regulate the generation rate, while *G*_*0*_ is the starting generation rate vector. *GP* is the generation probability that is employed to strike a balance between exploring and exploiting and is set with *GP* = *0.5*. Figure 1 shows the flowchart of the EO algorithm.

## Proposed algorithm

The standard equilibrium optimization (EO) algorithm suffers from two major problems: lack of population diversity and premature convergence. To overcome these issues, an enhanced version of EO is developed here with two main phases:

At the first step, the Opposition-Based Learning (OBL) technique is employed to improve population diversity^43^. In OBL, for each candidate solution X_i_ in the present population, an opposite solution X^'^_i_ is generated utilizing Eq. (13) where the upper and lower bounds of the search space are ${a}_{i}$ and ${b}_{i}$, respectively.

13 $$\mathop {X_{i} }\limits^{\prime } = a_{i} + b_{i} - X_{i}$$

The fittest solutions from the present population and the following generation are chosen from the opposing population. This helps in the exploration of the search space and avoids premature convergence.

Secondly, the Levy flight technique is applied to update the population. In Levy flight, the new solutions are generated by using a random walk process with a Levy distribution. The Levy distribution has an infinite variance and generates new solutions in large steps, which helps in the fast exploration process^44^. At each generation, a fraction of the best solutions (70%) is updated using Levy flight while the remaining solutions are updated using the traditional EO update equation. The Levy flight phase helps in escaping from local optima. New solutions are provided by utilizing the following random walk equation:

14 $$C_{new(i)} = C_{eq(i)} + Levy(D) \times S$$

Where *C*_*new(i)*_ and *C*_*eq(i)*_ are the new and old solutions, respectively. *S* is the step size and its value is adjusted by *S* = *1/t* and decreases over time, where *t* is the iteration number. This will make the steps larger at first, but decrease over time*. Levy(D)* is a Levy distribution and it is calculated as:

15 $$L\acute{e} vy \left(\beta \right)\sim 0.01\frac{u}{{\left|v\right|}^{1/\beta }}, u=\left(0, {\sigma }_{u}^{2}\right), v=\left(0, {\sigma }_{v}^{2}\right)$$

$${\sigma }_{u}={\left\{\frac{\Gamma \left(1+\beta \right)\text{sin}(\pi \beta /2)}{\Gamma \left(1+\beta /2\right)\beta {2}^{(\beta -1)/2}}\right\}}^{1/\beta }, {\sigma }_{v}=1$$

where *v* is a random value in a normal distribution. The *Levy* distribution has an infinite variance and generates new solutions in large steps, enabling fast exploration of the search space. The Pseudo-code of the introduced LOEO is shown in Algorithm 1.

### Computational complexity

The computational complexity of the proposed LOEO algorithm is obtained in this subsection. Computational complexity affects the algorithm's effectiveness, and in the presented LOEO algorithm, an algorithm with less complexity has been tried. Consequently, the complexity of the proposed method is expressed by the Big-O notation. The four primary factors that affect complexity are initialization, iteration count, fitness function assessment, and particle concentration updates. O (1) is the problem definition, and O (N × D), where D is the problem dimensions and N is the number of particles, is the complexity of the initialization phase. There are T iterations in total. Each particle's function evaluation complexity is O(C), and it takes O (N × C) time to assess the population's fitness. It costs O (N) time to save memory.

The complexity of the Opposition-Based Learning Operator is O (N × D), and the updating process takes O (N × D) time. Also, every iteration, the update process of particle's positions is performed for a number of population members, when M is the number of particles to which the Levy flight Operator is applied, has a complexity of O (M × D). As a result, the LOEO algorithm's overall temporal complexity is computed as follows.:

16 $$\text{O }\left(\text{LOEO}\right)=\text{ O }\left(1\right)+\text{ O }\left(\text{N}\times \text{D}\right)+\text{ O }\left(\text{T}\times \text{N}\times \text{C}\right)+\text{ O }\left(\text{T}\times \text{N}\right)+\text{O }\left(\text{T}\times \text{N}\times \text{D}\right)+\text{ O }\left(\text{T}\times \text{M}\times \text{D}\right)+\text{ O }\left(\text{T}\times \text{N}\times \text{D}\right)\cong \text{O}\left(\text{T }\times \left(\text{NC}+\text{MD}+2\text{ND}\right)\right).$$

**Algorithm 1:**
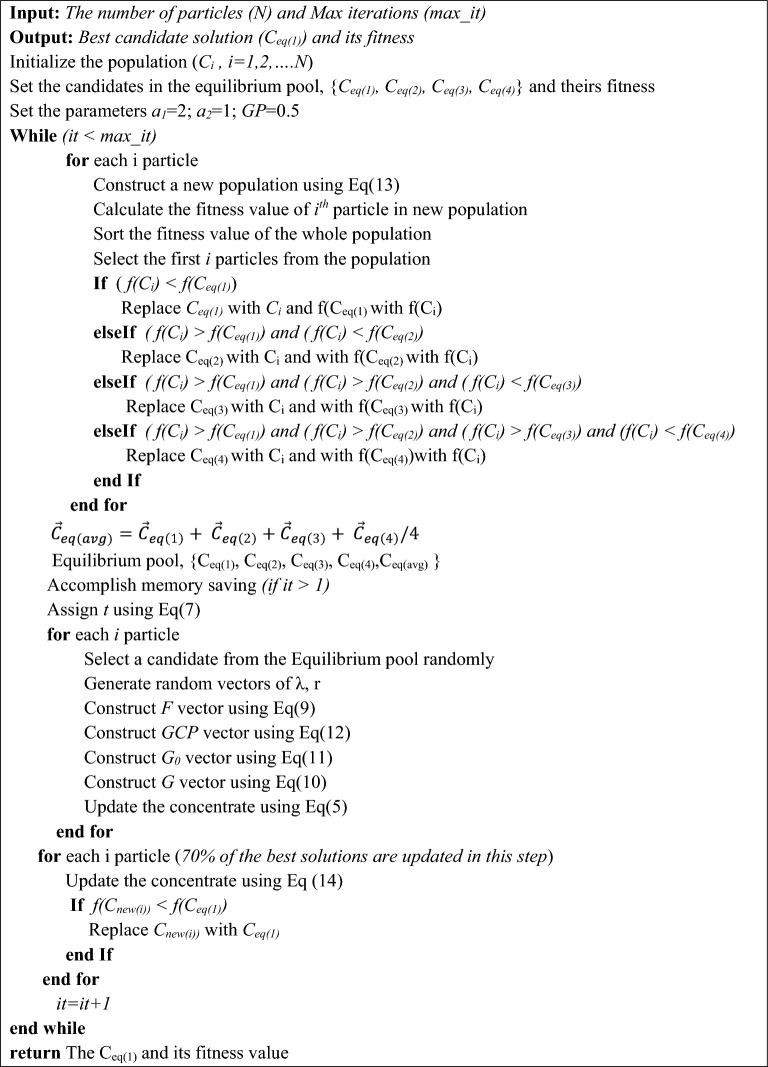
Pseudo-code of LOEO

### Application of LOEO in feature selection

This section measures the efficiency of the LOEO algorithm for binary optimization issues by applying it to the feature selection problem. Moreover, a binary adaptation of the original LOEO algorithm called BLOEO is developed to select the best features from the datasets of three data consisting of NSL-KDD, UNSW-NB15, and CIC-IDS2017.

The LOEO utilizes the variable threshold approach described in Eq. (17) to transform continuous solutions into binary representation in this section. The new binary position of the *i*th search individual is represented as ${b}_{i}^{d}\left(t+1\right)$, where *θ* is a variable threshold set by the user to 0.5.

17 $${b}_{i}^{d}\left(t+1\right)= \left\{\begin{array}{c}1\,\,\,\,\, if \,\,\,\,\,{C}_{i}^{d}\left(t+1\right)>\theta \\ \\ 0 \,\,\,\,\,if \,\,\,\,\,{C}_{i}^{d}\left(t+1\right)\le \theta \end{array}\right.$$

**Figure 2:**
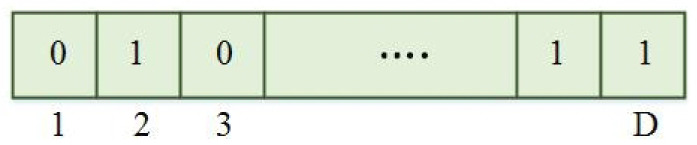
Solution representation for the feature selection problem.

The problem of feature selection is an optimization issue that involves binary variables. Each solution in this problem can be shown as a vector with one dimension, where the length of the vector determines how many features are present in the dataset. Each feature in the vector can take one of two values: "0" shows that the matching feature is not chosen, whereas "1" indicates that it is. A sample feature selection vector containing D features is represented in Fig. 2.

The fitness function employed in this problem has two primary goals, as stated in Eq. (18): to reduce the number of features chosen and maximize accuracy. The optimal solution obtains the most accuracy for classifier model while selecting the fewest possible features. To evaluate solutions, a KNN classifier is employed^45^. A subset of features is chosen by the solution for each iteration, and the KNN classifier trains data using the chosen feature subset and determine accuracy. As a result, the objective function is obtained as follows:

18 $${Fit}_{i}=\alpha E+\beta \frac{\left|{F}_{i}\right|}{D}$$

where *E* denotes the error rate of the KNN classifier, $\left|{F}_{i}\right|$ is the amount of the selected features in a subset of *F*_*i*_, and $D$ determines the whole of the features. $\alpha$ and $\beta$ are criteria to check the importance of accuracy and the number of features in the subset, respectively. In this paper, *α* = 0.99 and *β* = 0.01, based on^46^.

## Experimental results

In this section, the effectiveness of the BLOEO algorithm in identifying the better feature subset is examined on three datasets, including NSL-KDD, UNSW-NB15, and CIC-IDS2017. The results of our experiments are compared with other algorithms including the Sine Cosine algorithm (SCA)^47^, GWO^48^, HHO^49^, Differential Evolution (DE)^50^, and Salp Swarm Algorithm (SSA)^51^. To assess and contrast the proposed BLOEO algorithm with alternative methods, each algorithm is independently executed 20 times on a PC equipped with an Intel® 6.0 GB RAM Core™ i5 2.40 GHz processor. Additionally, Windows 10's MATLAB 2019b platform is used to run the apps.

### Datasets description

The NSL-KDD^52,53^, CICIDS2017^54,55^, and UNSW-NB15^56,57^ datasets are often utilized for evaluating network IDSs (NIDS). An upgraded version of the KDD Cup99 dataset is the NSL-KDD dataset, with duplicate records removed and the data size reduced. Simulated attacks include Denial of Service (DoS) attacks, User-to-Root (U2R) attacks, Remote-to-Local (R2L) attacks, and probe attacks. The CICIDS2017 dataset includes simulated real-world network traffic data and is divided into normal and attack behaviors, with attacks classified: brute force FTP, brute force SSH, DoS, heartbleed, web, infiltration, botnet, and DDoS attacks. The UNSW-NB15 dataset was constructed utilizing the PerfectStorm tool to simulate nine distinct network attacks, such as DoS, ShellCode, Worms, Fuzzers, and Backdoors, among others.

### Data preprocessing

In this section, the evaluated datasets are preprocessed in three main steps: data transformation, deletion of duplicate records, and data normalization^58^.

*Data transformation*: The data features consist of both numbers and strings. To apply the proposed method to the dataset, the string features need to be converted to numerical values.

*Deletion of duplicate records*: In the next step, duplicate records are removed from the dataset to prevent biasing the classifiers towards frequent records. At this stage, a large number of duplicate records are removed from the KDDCUP 99 dataset. The two datasets NSL-KDD and UNSW-NB15 have no duplicate records. Additionally, missing values are managed at this stage.

*Data normalization*: In the next step, data normalization is carried out. During the scaling process, the data values of each feature are placed in a proportional range. After scaling, the values of a feature are placed in the specified range [0, 1]. Equation (19) formulates the data normalization process in the range [0, 1]^59^.

19 $${X}_{normalized}=\frac{X- {X}_{min}}{{X}_{max}- {X}_{min}}$$

Finally, the feature selection process is applied to reduce the number of features of the dataset to increase the efficiency of classification. In this study, a wrapper-based feature selection method is proposed to reduce the number of dataset features using the proposed BLOEO algorithm.

### Parameter settings

For all experiments conducted, the KNN classifier in all methods with k = 5 to categorize feature subsets is employed to determine the optimal subset of features.

There are two reasons to choose KNN over other classifiers. Firstly, KNN is a simple yet powerful algorithm that can capture both linear and non-linear relationships within the data. This makes it well-suited for the exploratory nature of the feature selection task. Secondly, KNN requires minimal hyperparameter tuning, which aligns to maintain a lightweight and efficient evaluation process during optimization. This combination of effectiveness and efficiency makes KNN an ideal choice as the classifier for guiding the feature selection algorithm toward the optimal subset of features. To train the KNN, each dataset is split into K-folds for cross-validation purposes to assess the performance of the algorithms. To be more precise, the dataset is divided into K equal parts (K = 10) at random, K − 1 folds are utilized for training, while one-fold is reserved for the testing set. The algorithms are executed 20 times independently, using a uniform random distribution applied to create the starting population. Moreover, the maximum number of iterations and population size are set at 100 and 20, respectively, for all algorithms. When choosing the parameters for the KNN algorithm and each of the metaheuristic algorithms, a systematic adjustment process has been used to ensure computational feasibility. In this process, a balance is struck between model complexity and generalization performance on validation data, so algorithms can effectively explore the feature space without overfitting. Also, parameters were selected in optimization algorithms based on alignment with exploration and convergence characteristics. The algorithms' parameter settings are shown in Table 1.

**Table 1:** Parameters setting.

Algorithms	Parameters
GWO	A = [2,0] (Linearly decreasing)
DE	CR = 0.7
SCA	r_1_ = [2,0] (Linearly decreasing), r_2_ = [0, 2] r_3_ = [0, 2], r_4_ = [0, 1]
SSA	C1 = [2,0] (Linearly decreasing)
HHO	E = [2, − 2] → 0 (Linearly decreasing)
LOEO	a_1_ = 2, a_2_ = 1, GP = 0.5

### Evaluation metrics

The proposed BLOEO and comparative algorithms are evaluated based on various performance metrics, including fitness, the number of selected features, precision, accuracy, sensitivity or recall, specificity, and F-Score. The criteria were chosen to align with the main goal of this paper, which is to select crucial features and accurately predict network attacks. These criteria assess and evaluate the algorithm's performance from various perspectives and strive to strike a balance between algorithm complexity and performance. The definitions of these measures are computed using Eqs. (20)–(24)^60,61^. The numbers TP and TN in these equations represent the number of positive and negative samples, respectively, that the classifier correctly classifies. The number of positive samples that a classifier wrongly classifies as negative is represented by FN, and the number of negative samples that a classifier incorrectly classifies as positive data is represented by FP.

20 $$Accuracy= \frac{TP+TN}{TP+TN+FP+FN}$$

21 $$Sensitivity =\frac{TP}{TP+FN}$$

22 $$Specificity=\frac{TN}{TP+FN}$$

23 $$Precision=\frac{TP}{TP+FP}$$

24 $$\text{F}-\text{Score}=2*(\frac{Precision\times Recall}{Precision+Recall})$$

### Simulation results and discussion

The proposed binary LOEO algorithm's simulation results are reported in this subsection. On intrusion detection datasets. We analyze and discuss the findings by comparing the BLOEO model with state-of-the-art models.

#### Comparison of algorithms on the NSL-KDD dataset

To assess the LOEO algorithm's effectiveness in solving the feature selection problem, every experiment was carried out on three datasets including NSL-KDD, CICIDS2017, and UNSW-NB15. In Table 2, the BLOEO algorithm is compared with other competing algorithms regarding accuracy, fitness, and number of selected features, and the results are presented. All algorithms were executed 20 times and their average was calculated and reported in Table 2. The results of experiments on the NSL-KDD dataset show that regarding accuracy and fitness, even though all algorithms have relatively good results, compared to other competing methods, the BLOEO performs better. The classification accuracy of the proposed BLOEO algorithm with a value of 0.958 on NSL-KDD data is better than other algorithms. Also, this algorithm is superior to competing algorithms by obtaining values of 0.042 and 14.3 for Fitness and the number of selected features, respectively. This issue can have a positive effect on the quality of the IDS. After BLOEO, the SCA algorithm is ranked second and has a relatively good performance in selecting rich features in intrusion detection.

**Table 2:** Comparison results of algorithms in terms of accuracy, Fitness, and the number of selected features.

BLOEO	GOW	HHO	DE	SSA	SCA	Measures	Datasets
0.958	0.943	0.942	0.939	0.922	0.942	Accuracy	NSL-KDD
0.042	0.057	0.058	0.061	0.078	0.058	Fitness	
14.3	19.6	16.4	17.2	18.5	14.7	# Features	
0.945	0.896	0.889	0.903	0.863	0.891	Accuracy	CICIDS2017
0.055	0.104	0.111	0.097	0.137	0.109	Fitness	
18.4	23.5	24.7	27.9	25.5	24.1	# Features	
0.976	0.912	0.931	0.880	0.882	0.925	Accuracy	UNSW-NB15
0.024	0.088	0.069	0.120	0.118	0.075	Fitness	
10.8	13	11.7	14.6	18.8	12.6	# Features	

**Figure 3:**
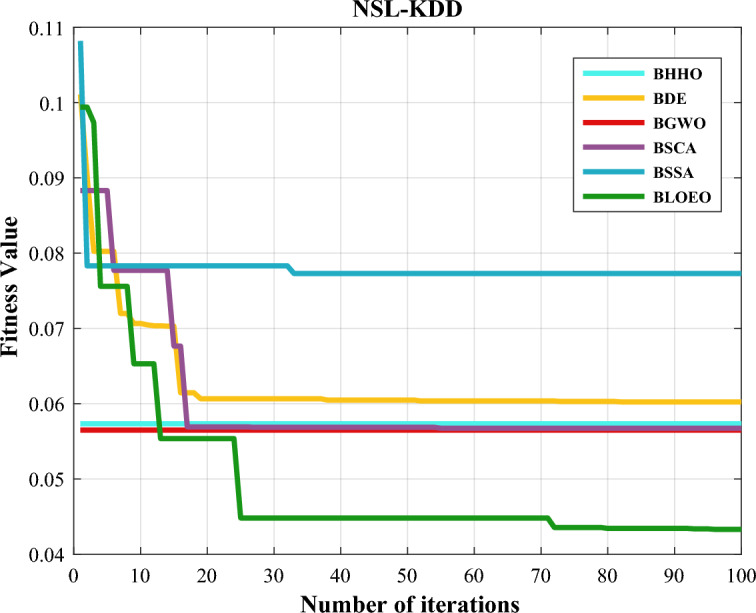
Comparison of convergence curve of algorithms on NSL-KDD dataset.

The convergence curve for the BLOEO and the other compared algorithms is exhibited in Fig. 3. This figure shows that all the algorithms are able to converge well to the optimal solution, but among the algorithms, the BLOEO algorithm is the best. Also, the BLOEO algorithm has been able to escape from local optima and converge to the global optimal solution, and compared to other algorithms, it has obtained the lowest fitness value.

#### Comparison of algorithms on the UNSW-NB15 dataset

**Figure 4:**
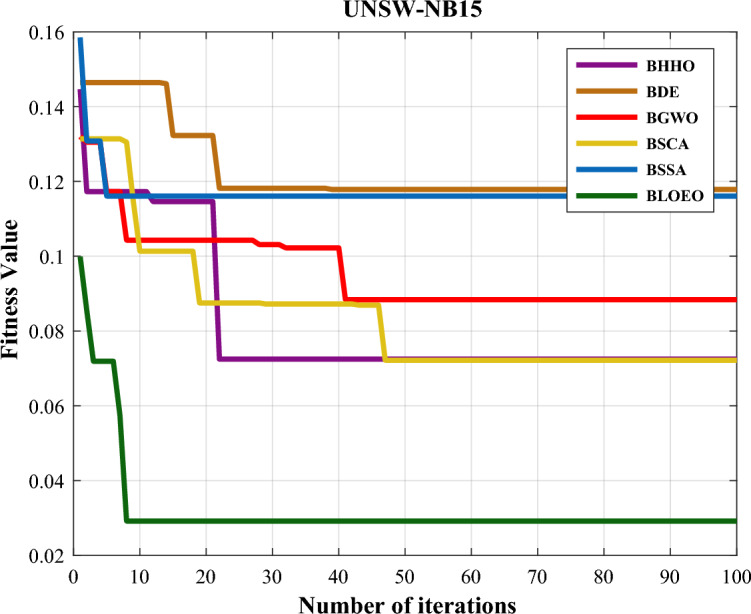
Comparison of convergence curve of algorithms on the UNSW-NB15 dataset.

The simulation results on the UNSW-NB15 dataset in Table 2 show the efficiency and the BLOEO algorithm's superiority over alternative methods. Numerical results regarding the accuracy criteria and the fitness of the compared algorithms show that the BLOEO algorithm can have an accurate and efficient diagnosis for possible attacks on the computer network with a good and significant difference in comparison to other algorithms. Figure 4 represents the convergence curve of metaheuristic algorithms along with the BLOEO algorithm. As represented in this figure, the BLOEO algorithm in the same initial iterations has been able to have a good convergence in reaching the optimal solution and obtain a relatively good convergence rate close to zero. In addition, by contrasting the number of selected features, we can understand that considering this criterion, BLOEO is almost equal to the SCA algorithm and is better than other algorithms. That is, it can obtain the desired classification accuracy by choosing fewer features. However, the comparison of metaheuristic algorithms with the proposed BLOEO algorithm is not limited to the criteria considered above. Experiments are also performed on the three introduced intrusion detection data sets regarding precision, sensitivity, specificity, and F-score. The numerical findings calculated from the tests are exhibited in Table 3. According to findings, the BLOEO algorithm has almost been able to perform better than others on all three data.

**Table 3:** Comparison results of algorithms in terms of precision, sensitivity, specificity, and F-score.

BLOEO	GOW	HHO	DE	SSA	SCA	Measures	Datasets
0.982	0.959	0.962	0.955	0.951	0.964	Precision	NSL-KDD
0.957	0.936	0.922	0.901	0.914	0.931	Sensitivity	
0.977	0.950	0.953	0.938	0.947	0.957	Specificity	
0.969	0.947	0.941	0.927	0.932	0.947	F-score	
0.971	0.933	0.912	0.932	0.892	0.922	Precision	CICIDS2017
0.954	0.900	0.890	0.911	0.869	0.901	Sensitivity	
0.966	0.924	0.898	0.925	0.887	0.920	Specificity	
0.962	0.916	0.900	0.921	0.880	0.911	F-score	
1.000	0.939	0.958	0.916	0.920	0.949	Precision	UNSW-NB15
0.973	0.918	0.936	0.934	0.911	0.956	Sensitivity	
0.983	0.928	0.944	0.908	0.918	0.931	Specificity	
0.986	0.928	0.946	0.924	0.915	0.952	F-score	

#### Comparison of algorithms on the CIC-IDS2017 dataset

**Figure 5:**
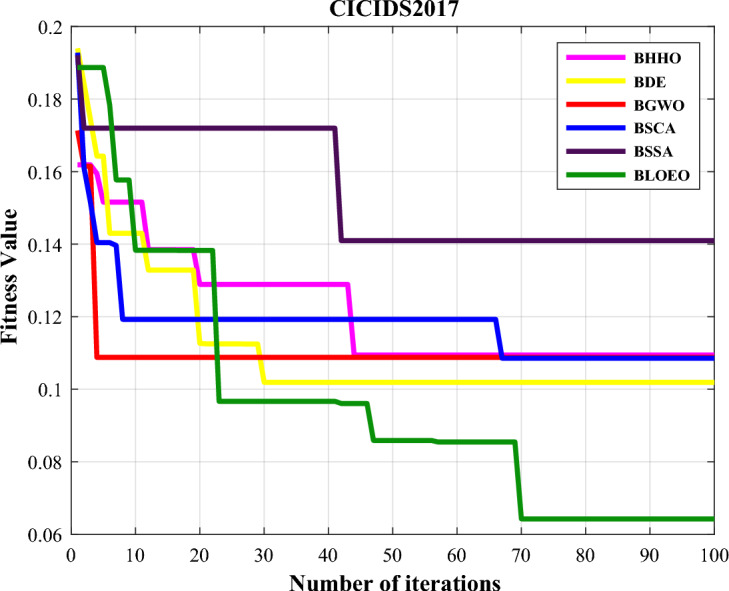
Comparison of convergence curve of algorithms on CICIDS2017 data.

The evaluation outcomes of various comparative algorithms and the BLOEO algorithm on the CICIDS2017 dataset are also represented in Table 2. In addition, Fig. 5 illustrates the convergence curve of all algorithms. According to this figure, The BLOEO algorithm has a good chance of escaping local optima and achieving convergence to the global best answer. Perhaps, if the number of iterations of the algorithm was more, it could still achieve better results by better searching the space. In general, the presented numerical results and the convergence diagram indicate that the BLOEO algorithm is more successful than other algorithms and has performed better in data classification and intrusion detection. Moreover, it has selected a few features from this data set and in this way creates an intrusion detection system with low complexity.

**Figure 6:**
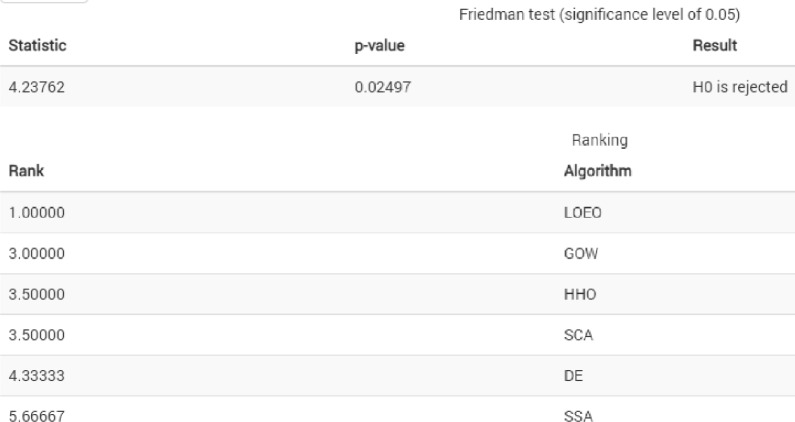
Friedman test results.

To show how the BLOEO algorithm performs in comparison to other algorithms, Friedman's statistical test can be employed to order. Figure 6 exhibits the findings of Friedman's test to compare the efficiency of the proposed algorithm and other competitors regards the fitness value of the algorithms. According to this figure, the BLOEO algorithm has been able to get the first rank among competing algorithms and they differ greatly from one another. Therefore, by proving this issue, the BLOEO algorithm applies to other optimization issues, especially binary problems such as feature selection.

Apart from the algorithms that were compared in the preceding section, the BLOEO algorithm was also compared with four state-of-the-art algorithms presented in recent years. These algorithms include BHOA^25^, BIMEO^31^, and the research is done by Tama et al.^62^, Alazzam et al.^38^ and Kareem et al.^63^.

Table 4 illustrates the evaluation findings of the compared algorithms on NSL-KDD, CICIDS2017, and UNSW-NB15. As observed, the BLOEO algorithm has the highest performance on the NSL-KDD dataset in terms of all the criteria considered in this table. In addition, BLOEO, BHOA, BIMEO, and GTO-BSA algorithm proposed by Kareem et al. are experimented on the CICIDS2017. The numerical results specified in Table 4 show that the accuracy, fitness and Specificity of the GTO-BSA are better than other algorithms with values of 0.987 and 0.013, respectively. Also, the Precision and Sensitivity of the BLOEO algorithm are better than other competitive algorithms. In order to compare the effectiveness of algorithms on the UNSW-NB15, the proposed algorithm has been contrasted with four introduced algorithms. The obtained numerical results show its superiority over competing algorithms.

**Table 4:** Comparison results of BLOEO algorithm with state-of-the-art algorithms.

Specificity	Sensitivity	Precision	#Features	Fitness	Accuracy	Methods	Datasets
0.957	0.932	0.964	16.7	0.059	0.941	BHOA	NSL-KDD
0.959	0.943	0.962	14.6	0.053	0.947	BIMEO	
–	0.637	–	37	0.218	0.782	Tama et al.^62^	
–	0.817	–	18	0.131	0.869	Alazzam et al.^38^	
0.973	0.913	–	14.7	0.045	0.955	Kareem et al.^63^	
**0.977**	**0.957**	**0.982**	**14.3**	**0.042**	**0.958**	**BLOEO**	
0.950	0.938	0.951	16.8	0.059	0.941	BHOA	CICIDS2017
0.934	0.914	0.939	17.9	0.078	0.922	BIMEO	
**o.996**	0.927	–	**10**	**0.013**	**0.987**	Kareem et al.^63^	
0.966	**0.954**	**0.971**	18.4	0.055	0.945	**BLOEO**	
0.981	0.970	**1.000**	11.6	0.025	0.975	BHOA	UNSW-NB15
0.983	0.973	**1.000**	12.3	0.027	0.973	BIMEO	
–	0.863	–	19	0.105	0.895	Tama et al.^62^	
–	0.897	–	14	0.087	0.913	Alazzam et al.^38^	
0.877	0.815	–	16.6	0.29	0.710	Kareem et al.^63^	
**0.983**	**0.973**	**1.000**	**10.8**	**0.024**	**0.976**	**BLOEO**	

Significant values are in bold.

## Conclusion and future works

This paper proposed an enhanced variant of the EO algorithm called BLOEO to select effective features for IDSs. The BLOEO algorithm utilizes opposition-based learning to enhance population diversity and a Levy flight mechanism to prevent local optima. The OBL helped the population explore a wider search space and escape from local optima. The Levy flight mechanism further improved the exploratory ability of the algorithm. Overall, the BLOEO algorithm provides an effective method for feature selection that can enhance the efficiency and scalability of IDSs. Experimental results on three datasets demonstrate that the BLOEO algorithm can drastically cut feature count while retaining good accuracy. Directions for future research include applying the BLOEO algorithm to other feature selection problems and datasets to further evaluate its performance.

## Data Availability

The datasets analyzed during the current study are available in the, https://ieee-dataport.org/documents/nsl-kdd-0, https://research.unsw.edu.au/projects/unsw-nb15-dataset, and https://www.unb.ca/cic/datasets/ids-2017.html. For the academic/public use of these datasets, the authors have to cite original papers.
